# Cardiac magnetic resonance imaging and follow-up of pacemaker events to identify the etiology and natural history of heart blocks

**DOI:** 10.1186/1532-429X-17-S1-P172

**Published:** 2015-02-03

**Authors:** Andres E Carmona-Rubio, Stefan Puchner, Ashley M Lee, Ting Liu, Godtfred Holmvang, Udo Hoffmann, Suhny Abbara, Umesh Sharma

**Affiliations:** Internal Medicine, University at Buffalo, Buffalo, NY USA; Cardiac CT/MRI/PET Imaging, Department of Radiology, Massachusetts General Hospital, Boston, MA USA

## Background

In patients with hemodynamically significant atrioventricular (AV) blocks of unknown etiology, cardiac MRI (CMR) with tissue characterization can provide incremental diagnostic information. Correlation of initial CMR findings with future events recorded by permanent pacemakers (PPM) can characterize the natural history of these serious and potentially life-threatening cardiac conditions.

## Methods

We studied a total of 78 patients with complete heart block, high-grade second degree heart block and symptomatic bundle branch blocks. In all patients, initial cardiac work-up was non-revealing for potential etiology, and CMR with late gadolinium enhancement (LGE) was performed. Following CMR, most of these patients underwent clinically indicated PPM placement. The interrogated PPM events were followed up for 1-4 years to monitor significant arrhythmias or long-term pacemaker dependence.

## Results

In patients with heart block, CMR identified a myocardial infiltrative process in 44%. In patients with symptomatic LBBB or RBBB, CMR identified a plausible etiology in approximately 25%. Presence of myocardial LGE by CMR had 90% sensitivity and 80% negative predictive value for long-term pacemaker dependence in patients with complete or high-grade AV blocks.

## Conclusions

In addition to the indications attributed in the current diagnostic algorithm, CMR can identify potential cause of hemodynamically significant AV-blocks in over 40% of the patients. Presence of LGE or other major diagnostic findings on CMR can uniquely identify patients with long-term pacemaker dependence.

## Funding

N/A.

**Figure 1:**
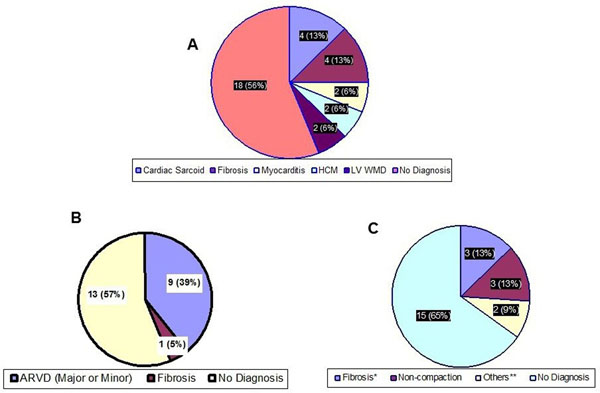
Pie Chart showing the relative distribution of the diagnostic categories of patients with- A) Complete or High-Grade AV-Block, B)RBBB, and C) LBBB.

**Figure 2:**
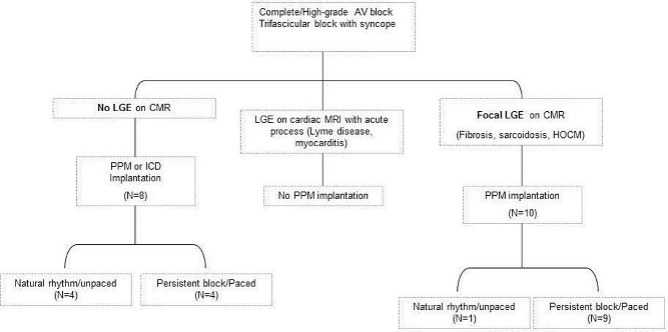
Pacemaker dependeance in patients with and without LGE or CMR.

